# Analysis of the Metabolites of Indole Degraded by an Isolated* Acinetobacter pittii* L1

**DOI:** 10.1155/2017/2564363

**Published:** 2017-12-13

**Authors:** Zuoyi Yang, Junhui Zhou, Yanbin Xu, Yaping Zhang, Haien Luo, KenLin Chang, Yujie Wang

**Affiliations:** School of Environmental Science and Engineering, Guangdong University of Technology, Guangzhou 510006, China

## Abstract

Indole and its derivatives are typical nitrogen heterocyclic compounds and have been of immense concern since they are known for the risk of their toxic, recalcitrant, and carcinogenic properties for human and ecological environment. In this study, a Gram-negative bacterial strain of eliminating indole was isolated from a coking wastewater. The strain was confirmed as* Acinetobacter pittii* L1 based on the physiological and biochemical characterization and 16S ribosomal DNA (rDNA) gene sequence homology. 400 mg/L indole could be completely removed within 48 h by the strain on the optimum condition of 37°C, pH 7.4, and 150 rpm. The organic nitrogen was converted to NH_3_-N and then to NO_3_^−^ and the organic carbon was partially transferred to CO_2_ during the indole biodegradation. The metabolic pathways were proposed to explain the indole degradation based on the liquid chromatography tandem mass spectrometry (LC-MS/MS) analysis of indigo, 4-(3-Hydroxy-1H-pyrrol-2-yl)-2-oxo-but-3-enoic acid, and isatin. The toxicity of the biodegradation products was evaluated using the Microtox test, which revealed that the metabolites were more toxic than indole. Our research holds promise for the potential application of* Acinetobacter pittii* L1 for NHCs degradation, production of indigoids, and soil remediation as well as treatment of indole containing wastewater.

## 1. Introduction

Indole and its derivatives, with highly toxic and carcinogenic properties, are mainly generated in large quantities as a result of industrial wastewater from pharmaceutical synthesis, fuel, cosmetics, pesticide, disinfectant, agrochemicals, and dyestuff and have recently gained wide attention [1–4]. These compounds also existed in large amounts of livestock manure emissions, which are serious pollution to the ecological environment with a sharp odor. Their heterocyclic structure makes them not only merely more soluble but also more difficult to degradation; therefore these cyclic compounds could be transformed through the soil and contaminated ground water.

Indole is a typical tryptophan metabolite in the natural environment, acting as plant hormone precursor and microbial signal molecule [5–7]. Furthermore, indole also acted as a gas pollutant because of its unfavorable odor, especially released from the pharmaceutical, coking, and livestock wastewater.

The technologies of contact glow discharge plasma degradation, photocatalytic degradation, and electro-Fenton oxidation were used to degrade indole, and the chemical oxidants such as chlorine and chlorine dioxide were used to control indole release [8, 9]. The photocatalytic degradation and chemical oxidation can efficiently break up indole, but the high investment and energy consumption confined the engineering applications, and the chemical oxidants could induce new toxic and carcinogenic compounds [8, 10, 11].

Some bacteria demonstrated their abilities of decomposing N-heterocyclic compounds. For example, a novel endophytic fungus* Phomopsis liquidambari* could catalyze indole into 2-aminobenzoic acid and 2-dioxindole and an isolated strain* Bacillus* sp. from petroleum-contaminated soil was able to decompose quinoline under aerobic conditions [12, 13]. Bacteria from genera* Alicycliphilus*,* Alcaligenes*, and* Thauera* were thought to be responsible for indole degradation [4]. Moreover, Qu et al. indicated that a rich set of oxidoreductases was expressed from a newly isolated* Cupriavidus *sp. SHE, which might be the most important factor for the efficient indole degrading [14].

It was reported that the C-N position of pyridine ring in indole and quinoline can be broken down by the microbe [12, 13]. One of the indole metabolic pathways was known as the isatin pathway, in which indole was degraded via the formation of indoxyl, 2,3-dihydroxyindole, isatin, N-formylanthranilic acid, anthranilic acid, salicylic acid, and catechol [15]. The other metabolic pathway was reported as the gentisate pathway, in which indole was degraded via indoxyl, isatin, anthranilic acid, and gentisate [16]. In this study, the metabolites were analyzed and the indole metabolic pathways of* Acinetobacter pittii* were illuminated.

The Microtox test was widely employed for evaluating the toxicity of the compounds and their products by using the prokaryote* Vibrio fischeri*. According to the Microtox test, Chen et al. indicated that the toxicity of the photoproducts of mefenamic acid were more toxic than their parent compounds [3]. Wang et al. analyzed the toxicity and mineralization of IDM under the conditions of AOPs; the results indicated that the inhibition rates of IDM declined dramatically in the presence of 1.0 g/L NCDs/g-C_3_N_4_ [17]. According to compounds, the types of degradation, and the toxicity assay methods, we can find out that the toxicity of different contaminants might increase or decrease in different occasion.

*Acinetobacter* spp. has been applied in a wide range of fields such as pharmaceutical industry and soil remediation. Researchers demonstrated that* Acinetobacter* spp. could utilize phenol or 4-nitroaniline as the sole carbon source and remove PAHs [18–21].* Acinetobacter* spp. was also reported to be able to degrade N-heterocyclic compounds [20, 22]. The effective biotechnology has been reported to optimize the indole removal rate [15, 22, 23]. The initial indole concentrations were usually controlled to be lower than 200 mg/L to prevent them from inhibiting the bacterial growth [14, 15].

In this study, we isolate a bacterial from a coking wastewater and investigate the degradation of indole; meanwhile, the metabolites were analyzed and the indole metabolic pathways of* Acinetobacter pittii* were illuminated and the toxicity changes about NHCs have also been detected.

## 2. Materials and Methods

### 2.1. Chemicals and Mediums

Indole (98.5%), quinoline (99.5%), and phenol (98%) were purchased from Aladdin Industrial Co., Ltd. Pyridine (99%) was purchased from Chengdu Kelong Chemical Reagent Co., Ltd. (Sichuan, China). High-performance liquid chromatography (HPLC) grade reagent methanol was obtained from Shanghai ANPEL Scientific Instrument Co., Ltd. (Shanghai, China). Ultrapure water from a Milli-Q apparatus (Smart2 Pure ultrapure water/water system integration, TKA, Germany) was applied in the HPLC and LC-MS/MS. All the other reagents were of analytic grade or above.

The mineral salt medium (MSM medium) was used for the isolation and biodegradation, which contained (g·L^−1^): K_2_HPO_4_, 0.8; KH_2_PO_4_, 0.2; CaCl_2_, 0.05; MgCl_2_·H_2_O, 1.07; FeCl_2_·H_2_O, 0.016; (NH_4_)_2_SO_4_, 1.0; NaCl, 5.0; pH 7.0. The Luria-Bertani medium (LB medium) contained (g·L^−1^): yeast extract, 5.0; peptone, 10; NaCl, 5.0; pH 7.0. All the media appending different indole concentration were autoclaved at 121°C for 30 min, and 2.0% (*w*/*v*) agar was added in the corresponding solid medium.

### 2.2. Bacterial Isolation and Identification

The target microorganism came from the sludge in a coke quenching effluent treatment system. The initial sludge was inoculated into 100 mL sterilized MSM containing 50 mg/L indole and then cultivated at 37°C with 150 rpm for 48 h. To get the indole-acclimating bacteria, 10% (*v*/*v*) of the above culture was transferred into the MSM with the addition of indole ranging from 50 to 400 mg/L every other five days for a month. Finally, the isolated strain L1 was characterized by morphological, physiological, and biochemical identification.

With the help of the BLAST software, 16S rDNA gene sequence was used to explore the homological relationship of the isolated strain L1. 16S rDNA gene of the strain L1 was amplified by PCR with universal primer pair 8F (5′-AGA GTT TGA TCC TGG CTC AG-3′) and 1492R (5′-GGT TAC CTT GTT ACG ACT T-3′). The phylogenetic tree of the isolated strain was inferred by MEGA 5.1 software using neighbor-joining method with a bootstrap value of 1000 replicates.

### 2.3. Evaluation of Indole Biodegradation

The strain L1 was cultivated in LB medium (37°C, 150 rpm, 24 h); the cells in logarithmic growth phase were collected and then centrifuged at 6000 ×g for 10 min (TDL-60B, Anting Scientific Instrument, Shanghai, China). Remove the medium and wash the pellet with PBS, and then resuspend the cell pellet to make OD_600_ = 1. The suspension was used immediately in the biodegradation experiments. To further investigate its capability of degrading indole, the removal rate at the different initial indole concentrations, temperatures, and pH values were analyzed by HPLC. The removal rates of pyridine, quinoline, and phenol, which are the typical compounds in the coking wastewater, were also tested when they acted as the sole carbon source in MSM medium inoculated* Acinetobacter pittii* L1. All samples were measured in triplicate. Moreover, the removal efficiency (RE) of indole was calculated with the equation RE (%) = (*C*_0_ − *C*_*t*_)/*C*_0_ × 100%, in which *C*_0_ and *C*_*t*_ are the initial and residual concentration.

### 2.4. Analytical Methods

The concentration of indole was detected by HPLC that contains a Shimadzu SPD-M20A photodiode array detector and two Shimadzu LC-20AD pumps (Shimadzu, Japan). The HPLC was equipped with a Zorbax Eclipse XDB-C18 column (2.1 mm × 150 mm, 3.5 *μ*m) and the mobile phase was composed of methanol : water (70 : 30 *v*/*v*) at 40°C, with the flow rate of 0.2 mL·min^−1^. Total organic carbon (TOC) was measured by Shimadzu (TOC-VCPH). UV spectrophotometer (UV-2100, Beijing Rayleigh) was utilized to determine the concentration of pyridine, quinoline, and phenol at 257 nm, 313 nm, and 280 nm, respectively. Ammonia nitrogen determination was detected by Nessler's reagent spectrophotometry.

In order to elucidate the possible indole degradation pathways, the metabolites of indole biodegradation were analyzed by LC-MS/MS, consisting of an Agilent 1100 series HPLC coupled to a 6410 triple quadrupole mass spectrometer (Agilent Technologies, USA).* Acinetobacter pittii* L1 was inoculated (5% *v*/*v*) into MSM including 200 mg/L indole and cultivated on the optimum conditions for 24 h. Then the culture was centrifuged at 6000 ×g for 10 min; the supernatant was filtered by 0.22 *μ*m micropolyether sulfone (PES) membrane syringe filter (Jinteng Experimental Equipment Co., Ltd. Tianjin). The solution was then transferred to a sample vial for characterization by LC-MS/MS. Separation was accomplished using an Agilent SB-C18 column (4.6 × 150 mm, 5 *μ*m). The eluent was made up of the ultrapure water (A) and methanol (B) at the flow rate of 1 mL·min^−1^. An autosampling device was employed to inject the sample which was detected at 270 nm. The elution was analyzed by the UV-vis detector of a mass spectral equipped with electrospray ionization (ESI) under negative mode. A mass full scan was conducted over a range of 50–550 *m*/*z* to identify the intermediates in the indole biodegradation, and the operation parameters were as follows: capillary voltage 3.5 kV, fragmentor 125 V, temperature 350°C, nebulizer pressure 30 psi, and nitrogen as the desolvation gas.

The samples were collected after every 3 hours of biodegradation of 100 mg/L indole solution, which was degraded by* Acinetobacter pittii* L1 at 37°C with 150 rpm for 24 h. Then Microtox Model DXY-2 was employed to determine the toxicity of samples and initial indole solution, which evaluates the ability of the metabolites to inhibit the bioluminescence of the strain* V. fischeri*. According to the equation* I* (%) = (*I*_0_ − *I*_*t*_)/*I*_0_ × 100%, the inhibition of* V. fischeri* can be calculated.

## 3. Results and Discussion

### 3.1. Microorganism Isolation and Identification

**Figure 1:**
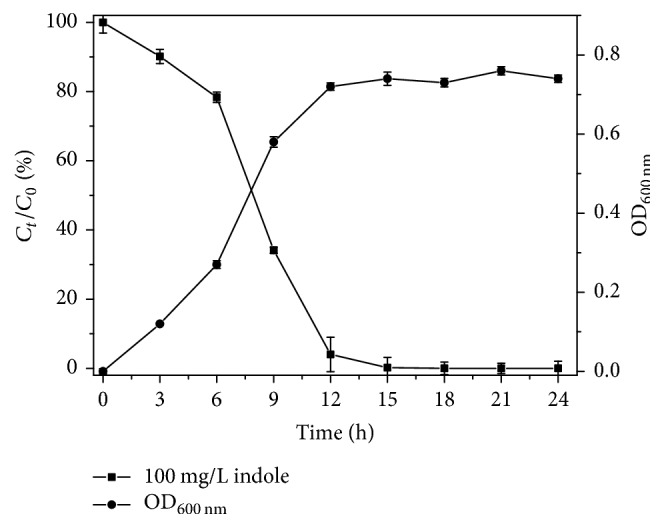
Growth curve of* Acinetobacter pittii* L1 and the change of indole concentration.

The strain L1 was isolated from the sludge in a facultative coke quenching tank. The growth curve of the strain and the indole remove rate in MSM were shown in Figure 1. 100 mg/L indole could be completely removed within 15 h, showing the better degradation efficiency compared with* Phomopsis liquidambari* (degrading efficiency 41.7%, 100 mg/L indole in 120 h) and* Pseudomonas aeruginosa* Gs (degrading 1.0 mM indole in 36 h) [13, 24]. As depicted in Figure 1, the bacteria were in logarithmic growth phase, OD_600_ increased with increasing biomass in 6–12 h. Therefore, the efficiency of indole degradation was improved dramatically.

**Figure 2:**
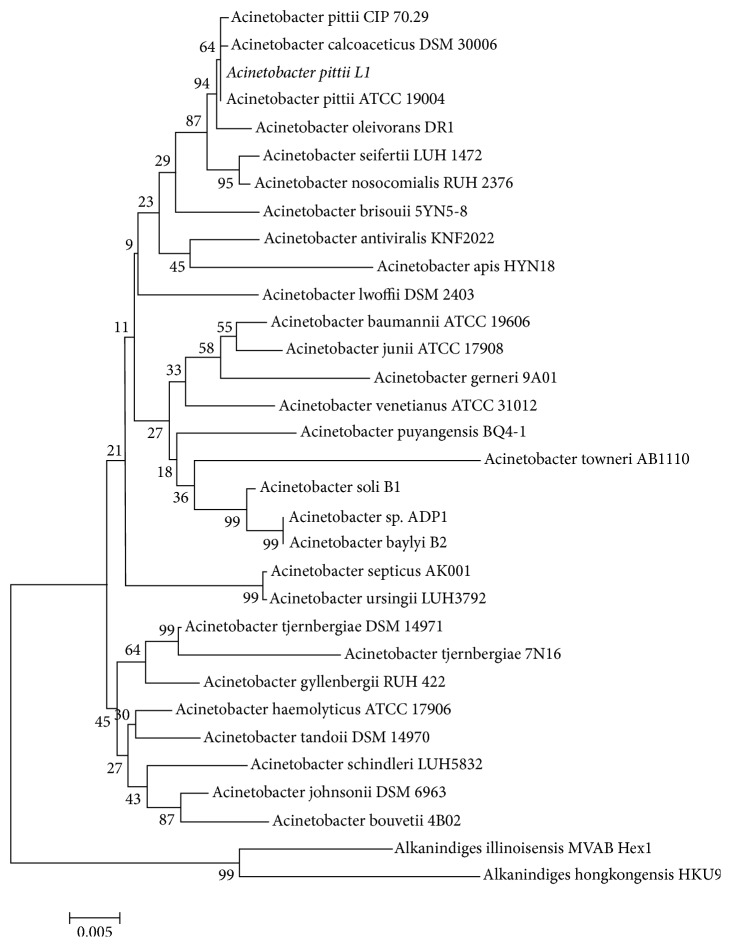
Phylogenetic tree of the strain L1 based on 16S rDNA analysis with the neighbor-joining method of the program MEGA 5.1. The numbers shown next to the nodes indicated the bootstrap values of 1000 for the confidence level.

The isolated strain L1 was a short rod-shaped, Gram-negative aerobe, and the physiological and biochemical properties were shown in Table 1. 16S rDNA gene sequence was applied to confirm the phylogeny relationships, and the strain was nominated as* Acinetobacter pittii* L1, which showed 99% gene sequence similarity with* Acinetobacter pittii* ATCC19004 (GenBank accession number: NR117621).  Figure 2 showed the phylogenetic tree correlated to the other species from the NCBI GenBank database by the method of neighbor-joining on the program MEGA 5.1.

### 3.2. Major Parameters Affecting the Biodegradation

#### 3.2.1. Initial Concentration

**Figure 3:**
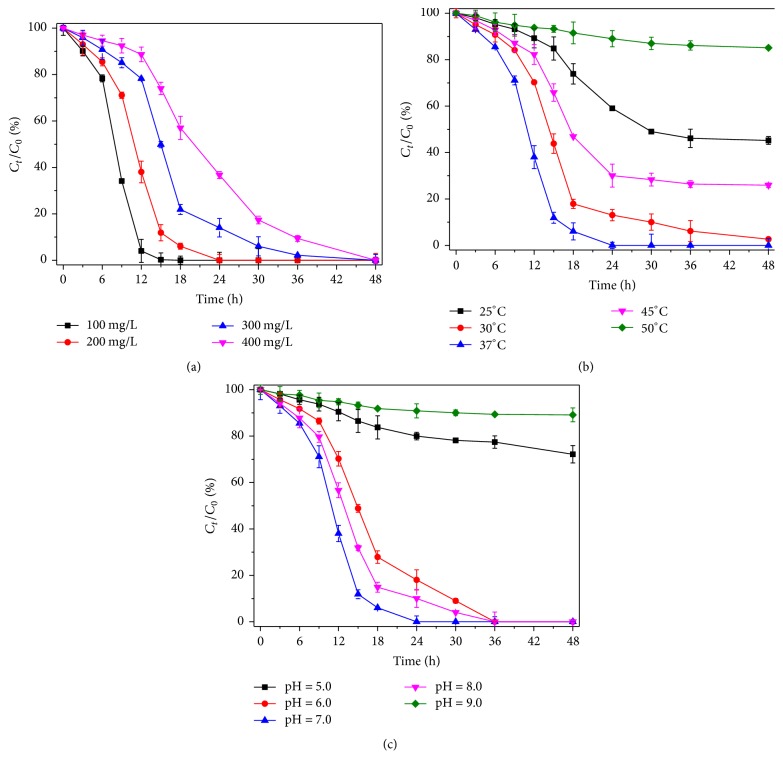
Different biodegrading efficiencies affected by the initial indole concentrations (a), temperatures (b), and pH values (c) in batch culture and the error bar value stand for the standard deviation of the triplicate.

When the initial indole concentration ranged from 100 to 400 mg/L, indole could be completely degraded within 48 h on the condition of 37°C, pH 7.0, and 150 rpm. As shown in Figure 3(a), the higher the initial indole concentration is, the smaller the removing rate of indole is. Qu et al. also reported the inhibited effect of indole to the stain SHE; for example, 50–100 mg/L indole could be completely degraded within 24 h, but 200 mg/L indole should be removed within 80 h [14].

#### 3.2.2. Temperature

The temperature was essential and sensitive to the microbe growth and the enzyme catalysis [25]. As shown in Figure 3(b), 200 mg/L indole was degraded by the strain L1 at pH 7.0 and 150 rpm for 48 h. The indole removal rates were determined as 54.81%, 97.36%, 100%, 74.07%, and 14.87%, respectively, at 25°C, 30°C, 37°C, 45°C, and 50°C. Therefore,* Acinetobacter pittii* L1 showed the optimum temperature to degrade indole at 37°C in this study, compared with white rot fungus at 25°C,* Phomopsis liquidambari* at 28°C,* Ps aeruginosa* and* Bacillus* sp. at 30°C [12, 13, 24, 26], and the fungus* Sporotrichum thermophile* at 45°C [23].

#### 3.2.3. pH Value

It was shown in Figure 3(c) that indole could be degraded by the strain L1 at pH 6.0, 7.0, and 8.0 at 36 h, and the indole removal rate was distinct at pH 7.0. Qu et al. [14] demonstrated indole could eliminate almost 90% by the strain SHE at pH 4.0–9.0. White rot fungus and* Ps aeruginosa* were reported to degrade indole at pH 5.0–9.0 [26, 27]. Besides,* Phomopsis liquidambari* showed the optimal efficiency to degrade indole at pH 4.5 [13].

### 3.3. Nitrogen Conversion

**Figure 4:**
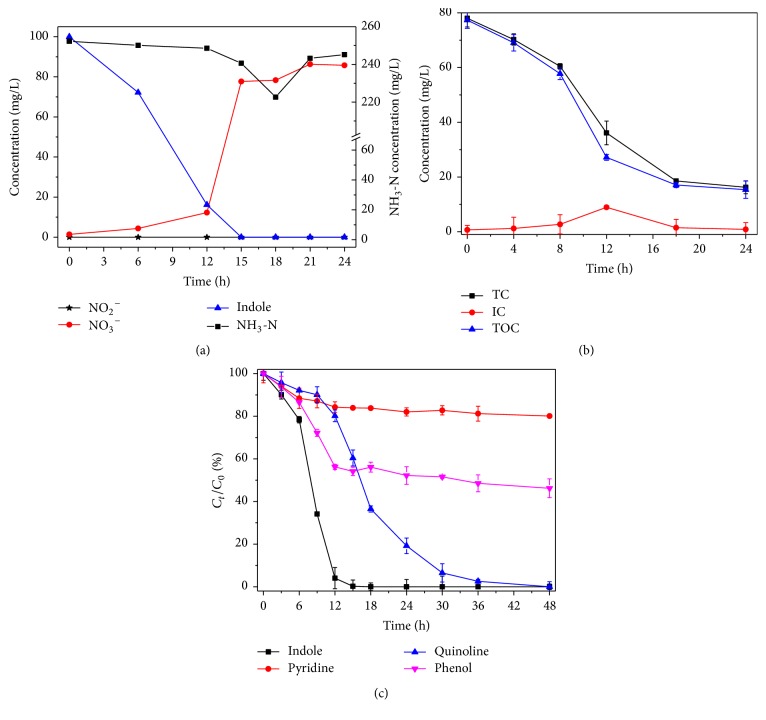
Organic carbon and nitrogen transformations during the indole degradation by* Acinetobacter pittii* L1, analyzed by the nitrogen source conversions (a), TC, IC, and TOC changes (b), and the different substrate degrading rates (c).

The changes of NH_3_-N, NO_3_^−^, and NO_2_^−^ were described in Figure 4(a) when 100 mg/L indole was absolutely removed within 24 h by the strain L1 in the MSM medium appended with 100 mg/L (NH_4_)_2_SO_4_. At the same time, the initial NH_3_-N decreased from 252.29 mg/L to 220.65 mg/L within 15 h, showing the microbial utilization of only about 31.64 mg/L. While the removal efficiency of indole was decreased, the concentration of NH_3_-N was increased after 15 h. Theoretically, NO_3_^−^ could be 90.13 mg/L (40.09 mg/L from ammonia nitrogen and 50.04 mg/L from indole), if all the nitrogen source was converted into NO_3_^−^. However, NO_3_^−^ was measured about 77.70~86.31 mg/L after 15 h, and NO_2_^−^ was not detected. According to Claus and Kutzner's reports, some nitrogen could be utilized to synthesize the intracellular substances [16]. Therefore, indole-N may be partially converted to NH_3_-N and then to NO_3_^−^ by* Acinetobacter pittii* L1.

### 3.4. Organic Carbon Changes

TC, TOC, and IC were investigated when 100 mg/L indole was biodegraded. As shown in Figure 4(b), the initial TOC 77.34 mg/L (similar to the theoretical value of 78.17 mg/L) was decreased to 15.37 mg/L at 24 h, but IC reached 8.94 mg/L at 12 h in the same way and decreased afterwards. It was demonstrated that some organic carbons were transferred to H_2_CO_3_ at first and then to CO_2_ during the indole biodegradation.  Figure 4(c) showed the removal efficiency of indole, pyridine, quinoline, and phenol by the strain L1. All the compounds could be degraded by* Acinetobacter pittii* L1 to some extent: indole and quinoline could be degraded completely within 48 h, 55.81% of phenol could be removed, and only 20% of pyridine was wiped over. Both indole and quinoline are structurally benzene-condensed heterocyclic compounds, but pyridine is a monocyclic compound. It was shown that the strain L1 was good at degrading condensed heterocyclic compounds, presumably because of the crucial metabolizing enzymes in the microbe.

### 3.5. Analysis of the Metabolic Pathways

The metabolites of blue, pink, and virescent hues were visible during the indole biodegradation by* Acinetobacter pittii* L1. LC-MS/MS was performed to detect the metabolites, the prominent molecular ion [M-H]^−^ peak at *m*/*z* 260.7, 180.0, and 145.9 of the intermediates was, respectively, shown at the retention time of 2.2 min, 4.5 min, and 2.1 min, and their formulas and major mass fragmentation values were listed in Table 2.

**Figure 5:**
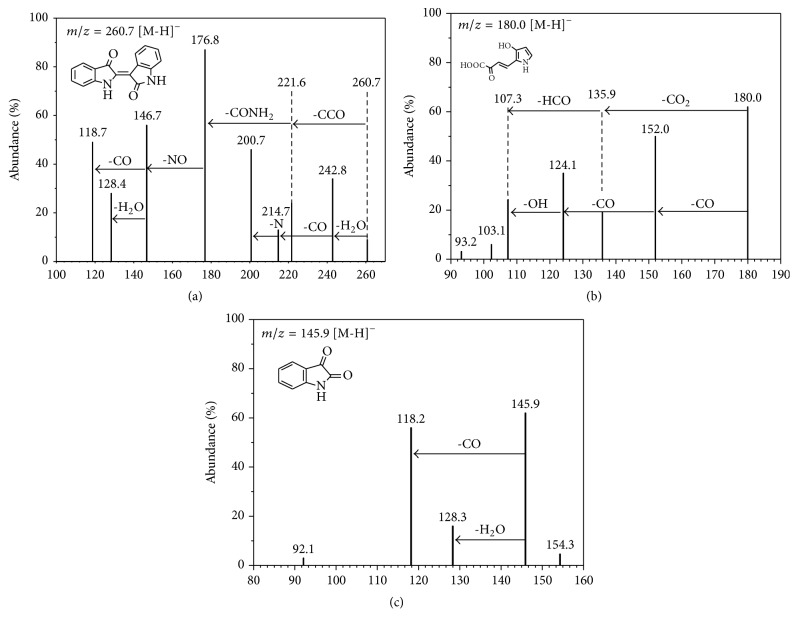
Fragment analysis of the secondary ion mass spectrometry of all the metabolite.

Figure 5(a) showed the deprotonated molecule at *m*/*z* 260.7 by the full scan analysis of the samples; the ions scan revealed the major mass spectra fragments at *m*/*z* 242.8 (−18), 221.6 (−39), 214.7 (−46), 200.7 (−60) 176.8 (−84), 146.7 (−114), and 118.7 (−142). The analysis revealed that the fragmentation ions at *m*/*z* 242.8, 176.8, 146.7, and 118.7 corresponded to the losses of 18, 44, 30, and 28 Da, showing the losses of H_2_O, CONH_2_, NO, and CO, respectively, from the parent deprotonated molecule. Considering the molar mass of 262 Da, the losses of CO, CONH_2_, NO, and H_2_O from the parent deprotonated molecule, the metabolite of indigo (C_16_H_10_N_2_O_2_) was confirmed.

Figure 5(b) showed the deprotonated molecule at *m*/*z* 180.0 by the MS^2^ fragmentation spectrum. Four major fragment ions of carboxide (CO), carboxylation (CO_2_), aldehyde (CHO), and hydroxide (OH) could be speculated, according to the mass losses of 28 Da at *m*/*z* 152.0 and 124.1, 44 Da at *m*/*z* 135.9, and 29 Da and 17 Da at *m*/*z* 107.3. Fukuoka et al. reported that a pyrrole ring could be contained in such compound as the MS^2^ fragmentation spectrum of ions at *m*/*z* 180.0 [28]. Therefore, the intermediate was proposed to be the 4-(3-hydroxy-1H-pyrrol-2-yl)-2-oxo-but-3-enoic acid according to the analytical data and the molecular formula of C_8_H_7_NO_4_.

During the indole biodegradation, a pink compound briefly appeared less than 4 h, which was detected in the MS^2^ fragmentation spectrum of ions at *m*/*z* 145.9 (Figure 5(c)). Additional ion fragments at *m*/*z* 118.2 (−28) and 128.3 (−18) indicated the isatin mass losses of 28 Da as CO and 18 Da as H_2_O, respectively. Qu et al. indicated that indoleoxide, hydroxyindole, or 2,3-dihydroxyindole was the metabolites from the aerobic biodegradation of indole and then was oxidized to isatin immediately [14]. Because of the mass losses of (−28) and (−18) detected in the MS^2^ fragmentation spectrum of ions at *m*/*z* 145.9, this metabolite might be derived from the carboxide and hydroxyl additions. Similarly, Zou and Koh observed the same mass losses with the determination of indigotin by LC/ESI-MS/MS at *m*/*z* 242.8, but the mass loss means the derivation of carboxide addition and phenolic hydroxyl [29].

**Figure 6:**
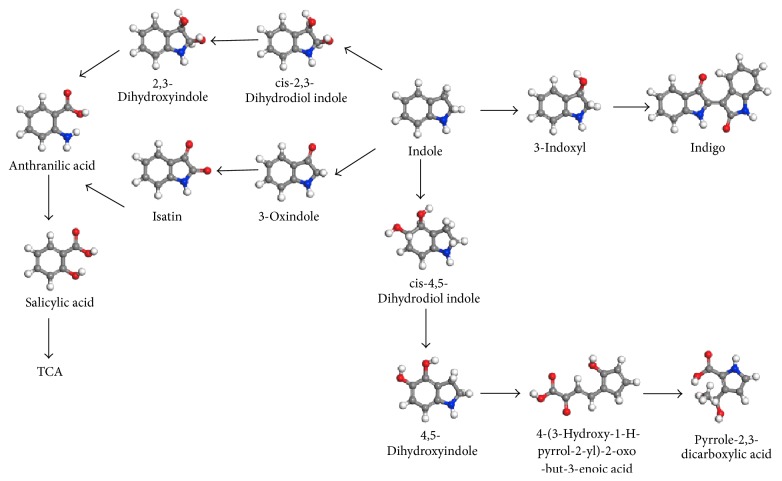
Speculated indole metabolic pathway degraded by* Acinetobacter pittii* L1. The gray, white, blue, and red balls are carbon, hydrogen, nitrogen, and oxygen atoms, respectively.

Nevertheless, other familiar metabolites reported such as indoxyl, oxindole, anthranilate, salicylate and gentisic acid, and hydroxyindole in similar researches were not observed [14]. Therefore, the metabolic pathways of indole degradation by* Acinetobacter pittii* L1 were speculated (Figure 6), based on the analysis of the identified metabolites in this study.

### 3.6. Analysis of the Toxicity

**Figure 7:**
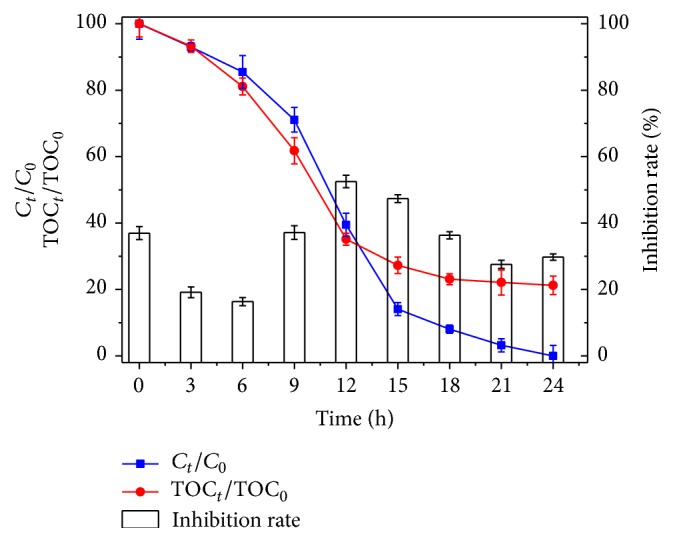
Variation of toxicity by Microtox test, inhibition rate, indole concentration, and TOC during degradation process.

To elucidate the ecological risk of the biodegradation products of indole, it is necessary to determine the toxicity evolution of indole contaminated, and the luminescent bacteria* Vibrio fischeri *were used to assess the changes of the acute toxicity of the compound.  Figure 7 represents the inhibition rate, biodegradation rate, and TOC value of 100 mg/L indole solution fewer than 24 h of biodegradation. As depicted in Figure 7, the initial inhibition rate of indole was 36.97% based on the above equation.

As 14.51% indole was degraded, the* Vibrio fischeri* inhibition rate decreased dramatically to 16.34%. Subsequently, the inhibition rate was quickly increased to 52.49% when a ~60.53% degradation rate of indole was obtained. It demonstrated the generation of more toxic products during the degradation of indole. Then the inhibition rate declined dramatically in the second 12 h. The inhibition rate reached an increasing tendency, which means that a new metabolite may be produced in the last. But the final inhibition (28.93%) was less than the initial inhibition (36.97%), which means that the metabolites products of indole were ultimately less toxic than the parent compound indole.

In general, the majority of indole was metabolized into intermediate products without the complete mineralization of CO_2_ and H_2_O. Therefore, the ecological risk of indole in engineered systems and ambient aquatic environments must be more closely scrutinized.

## 4. Conclusions

Indole and its derivatives are the representative compounds of NHCs, which are difficult degradable organic pollutants with lethal effect on ecological environment and characteristic odor. An efficient indole degrading* Acinetobacter pittii *L1 was isolated from a coking wastewater, 400 mg/L indole in 48 h, which could be thoroughly degraded in the optimum conditions. The removal efficiency of indole decreased after 15 h, but the concentration of NH_3_-N and NO_3_^−^ increased, showing the change of indole-N, and some organic carbon of indole was likely transferred into CO_2_ eventually, and the metabolites products of indole were ultimately less toxic than the parent indole. It is shown that the isolated* Acinetobacter pittii* L1 could eliminate indole effectively and the metabolic pathways were speculated by the analysis of metabolites. Our results elucidated that the high efficiency of degradation ability to degrade indole makes* Acinetobacter pittii *L1 as an excellent candidate for producing indigo and eliminating NHCs and for printing and dyeing industrials with indole contaminated area.

## Figures and Tables

**Table 1:** Biochemical characteristics of* Acinetobacter pittii* L1.

Biochemical and culture conditions	Results
Gram-staining	−
Anaerobic growth	−
Glucose utilization	−
Catalase	+
Oxidase	−
Citrate utilization	+
V-P test	−
Gelatin liquefaction	−
Hydrogen sulfide test	−
Hydrolysis of starch	−
MR test	−

+: positive reaction; −: negative reaction.

**Table 2:** LC-MS/MS data of the retention time, formula, and major mass fragmentation values to identify the indole biodegrading metabolites.

Parent ion	*t*_*r*_ (min)	Formula	Major mass fragmentation value *(m/z)*	Compounds
[M-H]^−^				
260.7	2.2	C_16_H_10_N_2_O_2_	ES-: 247.1/242.8/221.6/214.7/200.7/176.8/146.7/118.7	Indigo
180.0	2.1	C_8_H_7_NO_4_	ES-: 152.0/135.9/124.1/107.3	4-(3-Hydroxy-1H-pyrrol-2-yl)-2-oxo-but-3-enoic acid
145.9	4.5	C_8_H_5_NO_2_	ES-: 128.3/118.2	Isatin
